# The Quest to Enhance the Efficacy of Berberine for Type-2 Diabetes and Associated Diseases: Physicochemical Modification Approaches

**DOI:** 10.3390/biomedicines8040090

**Published:** 2020-04-18

**Authors:** Solomon Habtemariam

**Affiliations:** Pharmacognosy Research Laboratories & Herbal Analysis Services UK, University of Greenwich, Chatham-Maritime, Kent ME4 4TB, UK; s.habtemariam@herbalanalysis.co.uk; Tel.: +44-208-331-8302

**Keywords:** nanoparticles, efflux, bioavailability, pharmacokinetics, synthesis, efficacy

## Abstract

Berberine is a quaternary isoquinoline alkaloid that has been isolated from numerous plants which are still in use today as medicine and herbal supplements. The great deal of enthusiasm for intense research on berberine to date is based on its diverse pharmacological effects via action on multiple biological targets. Its poor bioavailability resulting from low intestinal absorption coupled with its efflux by the action of P-glycoprotein is, however, the major limitation. In this communication, the chemical approach of improving berberine’s bioavailability and pharmacological efficacy is scrutinised with specific reference to type-2 diabetes and associated diseases such as hyperlipidaemia and obesity. The application of modern delivery systems, research from combination studies to preparation of berberine structural hybrids with known biologically active compounds (antidiabetic, antihyperlipidaemic and antioxidant), as well as synthesis approaches of berberine derivative are presented. Improvement of bioavailability and efficacy through in vitro and ex vivo transport studies, as well as animal models of bioavailability/efficacy in lipid metabolism and diabetes targets are discussed.

## 1. Introduction

Type 2 diabetes (T2D) is a classic example of chronic metabolic disorder which is characterised by consistently high levels of blood glucose, mainly due to lack of sensitivity to insulin and often combined with some degree of pancreatic β-cell dysfunction. The global emergency of T2D is highlighted by the WHO with the latest data showing the rise in global prevalence of diabetes among adults over 18 years of age from 4.7% in 1980 to 8.5% in 2014 [1]. The data also shows the number of people with diabetes has risen from 108 million in 1980 to 422 million in 2014. Estimates for the year 2019 also revealed that about 463 million adults (20–79 years) were living with diabetes with a further projection of 700 million sufferers by the year 2045 [2]. The stark reality of the epidemic proportion of the rise in obesity, the major risk factor for diabetes, is even more alarming. The WHO fact sheet for the year 2016 shows that more than 1.9 billion adults, 18 years and older, were overweight [3]. Of these, over 650 million were obese. This data corresponded to 39% of adults aged 18 years and over who were overweight in 2016, of which 13% were obese [3]. It remains the case that there is no drug or cure for T2D and associated diseases, although drug therapies and lifestyle changes could reduce both their incidence and morbidity. In view of natural products being reliable sources of medicine, the search for novel drugs with a lower toxicity profile continues. There is also a drive for exploitation of medicinal foods, which have a profound effect on both T2D and obesity [4].

**Figure 1:**
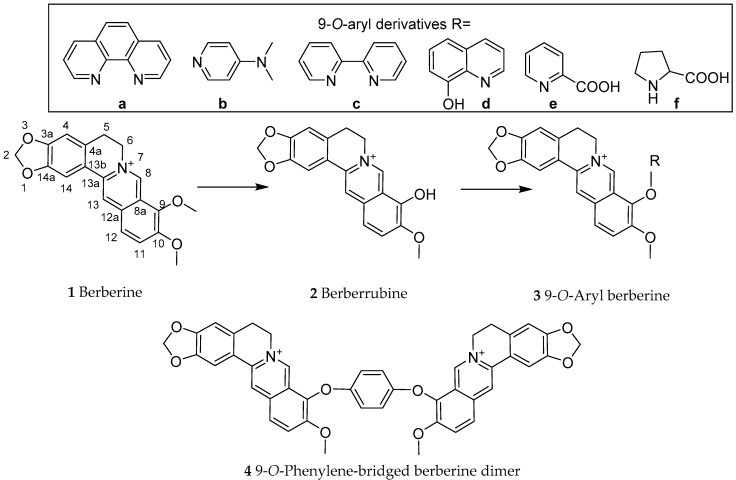
Berberine dimer formation via copper-catalysed aryl-*O*-coupling synthesis approach [34].

Berberine (**1**, Figure 1) is one of the best characterized natural compounds with diverse pharmacological effects, including anticancer [5,6,7], anti-inflammatory [8,9], cardioprotective [10,11] and enhancement of memory function [12,13,14] effects. Its antidiabetic [15,16,17], antiobesity and anti-hyperlipidaemic [16,18] effects have also been well established. Berberine induces its effects in diabetes and associated diseases through diverse effects including inhibition of gluconeogenesis and lipid accumulation both in cellular and animal models via modulating key signal transduction pathways. For example, it can suppress the expression of hepatocyte nuclear factor-4α (HNF-4α) and the microRNA miR122, which are linked to lipid and glucose metabolism [19]. As a major source of berberine, the stems and roots of *Berberis aristata* [20,21], *B. darwinii* [22,23], *B. Petiolaris* [24] and *B. vulgaris* [25] have been extensively investigated. Numerous other high berberine-yielding medicinal plants have further been reported with the most famous being *Coptis chinensis* (Chinese goldthread) and related species and *Hydrastis Canadensis or* goldenseal. Since berberine acts through the polypharmacology principle of drug action [26], there is a growing interest to increase its efficiency through synthetic derivatives that have better pharmacokinetics and pharmacodynamics profile. With the aim of promoting research in this field, our recent communication scrutinised the gut microbiota as a hidden therapeutic target that dictate the diverse pharmacology of berberine [27]. By using berberine as an inspiring lead natural product, recent attempts to enhance its anticancer therapeutic efficacy through semisynthetic approaches have also been reported [28]. In continuation of this effort, recent advances in the physicochemical modifications of berberine to target T2D and associated diseases is scrutinised herein.

## 2. Chemical Synthesis of Berberine

The synthesis of berberine (1) has been described by many authors. In their pioneering work in 1969, Kametani et al. [29] described the synthesis of berberine iodide with very low yield and multiple steps. Since then, several studies have shown improvement in the total synthesis approach with a yield of 50% and a 5-step procedure reported by Gatland et al. [30]. This was achieved by using a flexible palladium-catalysed enolate arylation as the key step through silver nitrate-catalysed aromatic annulation of *O*-(1-alkynyl) arylaldehydes and ketones with ammonium acetate. The synthesis of berberine by Reddy et al. [31] was commended as a mild and efficient protocol. A robust four- to eight-step synthesis that allowed the synthesis of over 50 protoberberine alkaloids has also been described by Zhou and Tong [32]. Overall, a yield of up to 50% with up to 5 steps of synthesis appears to be a productive approach for berberine synthesis. A compromise between reduction in the number of steps involved and yield has also been made in the synthesis approach [33]. The total synthesis of berberine analogues such as epiberberine with overall yield of 26.1% has further been described [34].

Most of the chemical modification attempts include the conversion of berberine through demethylation reaction to berberrubine (**2**). In fact, the pyrolysis of berberine can be done at high temperature under vacuum to generate the 9-hydroxyl derivative (berberrubine, **2**). As part of improving bioavailability by adding lipophilic substituents, Teng et al. [35] employed a copper-catalysed aryl-*O*-coupling synthesis approach to introduce a range of phenyl substituents (**3a–f**) as well as the phenylene-bridged berberine dimer (**4**) (Figure 1). The bioavailability of these derivatives is yet to be explored. The design and synthesis of berberine dimer as a fluorescent ligand with high affinity towards G-quadruplexes has also been described [36]. Further chemical approaches to berberine chemistry including its synthesis has been reviewed by Nechepurenko et al. [37].

## 3. General Pharmacokinetics Approach

### 3.1. Improving Berberine Bioavailability

**Figure 2:**
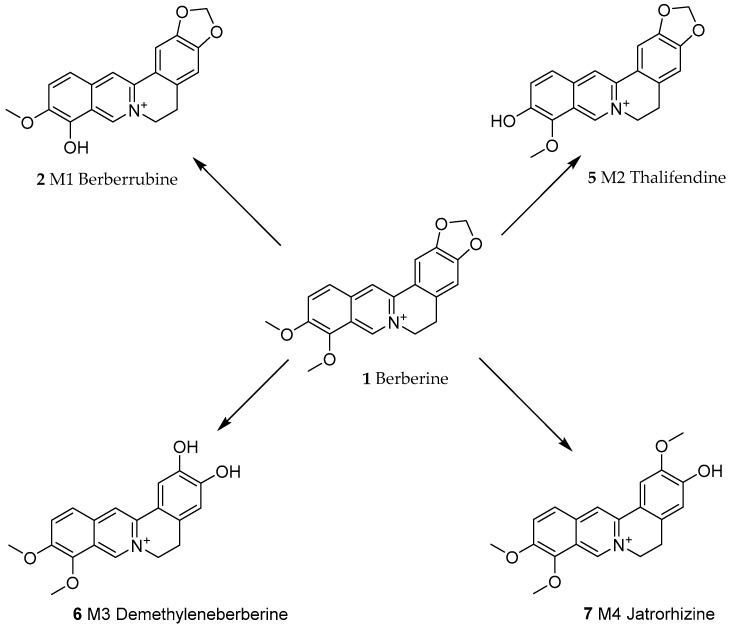
Phase-I transformation of berberine by CYP450 (Cytochrome P450) in liver cells [38]. CYP2D6 and CYP1A2 play a major role in transforming berberine into M2; CYP2D6, CYP1A2 and CYP3A4 are key to M3 production. M1 and M4 could be below detection level.

An incredible amount of research effort has been invested in understanding the rather low bioavailability of berberine. In rats, berberine has been shown to undergo intestinal first-pass elimination, while absorbed berberine is heavily distributed and degraded in hepatic tissues [38]. The extensive metabolism of berberine by hepatic cytochrome P450 enzymes such as CYP2D has been demonstrated both in mice and humans [39]. While CYP enzymes are also active in the degradation of berberine in intestinal tissues, their extensive role in the phase-I transformation of berberine in the liver has been established. In this case, Li et al. [40] identified four major metabolites: berberrubine (M1, **2**), thalifendine (M2, **5**), demethyleneberberine (M3, **6**) and jatrorrhizine (M4, **7**) (Figure 2). Furthermore, berberine undergoes intestinal oxidative demethylation followed by glucoronidation [41]. Another widely researched topic related to berberine’s low bioavailability is its efflux back to the intestinal lumen by the action of permeability glycoprotein (P-glycoprotein, P-gp) [42,43]. On these bases of poor absorption, efflux and extensive metabolism, the absolute bioavailability of berberine is far less than 1%. Accordingly, one of the approaches for improving berberine’s efficacy is through formulation primarily via improvement of its bioavailability from the gut.

Stressing the CYP2D6 and CYP3A4-mediated intestinal metabolism as one major factor for berberine’s poor bioavailability, Khan et al. [44] prepared a poly lactic-co-glycolic acid (PLGA) nanoparticles by encapsulating berberine with PLGA-doxorubicin conjugate (PDC). While the cytotoxicity in breast cancer cells (MDA-MB-231 and T47D lines) in vitro and in vivo in rats could be enhanced by up to 14-fold, the preparation has a far better transport in cultured Caco-2 cells and rat intestinal perfusion preparations due to improved permeability (5.5-fold) and reduced P-gl efflux (2-fold). In view of the antihypercholesterolaemic effect of berberine, Ochin and Garelnabi [45] also prepared berberine nanoparticles by encapsulating with PLGA-poly(ethylene glycol) (PEG). In cellular models using HepG2 cells, targeting proprotein convertase subtilisin/kexin type 9 (PCSK-9) by this nanoparticle preparation to lower low-density lipoprotein cholesterol (LDL-C) has been presented. Nanoemulsion-based delivery systems with a significant increase in intestinal permeability as assessed by Caco-2 cell monolayers and a significant reduction in efflux have also been shown by Li et al. [46]. The study by Xie et al. [47] further showed that berberine nanoparticles were effective in ameliorating ischemia-reperfusion injury in renal tubular epithelial cells. The study examined the dual-functional Brij-S20-modified nanocrystal formulation which was shown to enhance the intestinal transport and oral bioavailability of berberine [48]. As a mechanism of action in MDCK-MDR1 cells, a reversible modulation on P-gp function accompanied by a marked increase in P-gp mRNA expression but without significant influence on the P-gp protein expression were shown.

Berberine-loaded chylomicrons with 10.5-fold enhancement in permeability in ex vivo intestinal permeability studies and about 2-fold increase in Caco-2 cell model have been shown by Elsheikh et al. [49]. A further study by the authors also characterised a novel berberine-loaded cremochylomicrons primarily for targeting lymphatic system through oral dosage [50].

Zhou et al. [51] synthesised a novel berberine-loaded chitosan microsphere as a means of sustained release, increased bioavailability and with better enhancement potential of anti-apoptotic effect induced by sodium nitroprusside. This, along with other osteoarthritis markers in these cells, was suggested as a mechanism of berberine’s increased pharmacological efficacy through such formulation. By using the conjugate of chitosan/fucoidan and taurine, Wu et al. [52] prepared berberine nanoparticles for treatment of defective intestinal epithelial tight junction barrier induced by lipopolysaccharides (LPS). In a Caco-2 cells/RAW264.7 cell co-culture system, intestinal epithelial tight junction disruption (tight junction ZO-1 protein) could be ameliorated by the preparation.

Zhu et al. [53] presented a self-microemulsifying berberine hydrochloride preparation with improved relative bioavailability in vivo by about 2.42-fold. On the other hand, Zhang et al. [54] prepared a solid dispersion composed of berberine-phospholipid complex (BPC), d-α-tocopheryl polyethylene glycol 1000 succinate (TPGS 1000) and SiO_2_ with a resulting up to 2.0-folds increased absorption in a single-pass intestinal perfusion study. It also increased the relative oral bioavailability of berberine by 322.66%. Novel injectable Lu et al. [55] and skin penetrating [56] berberine have also been prepared. The significance of these formulations to diabetes and related metabolic disease are shown in the following sections.

### 3.2. Berberine Improving the Pharmacokinetics of Other Drugs

The in vivo (rats) drug–drug interactions between lovastatin and berberine was studied using an UPLC-MS/MS method [57]. The area under curve (AUC) and maximum/peak serum concentration (C_max_) could be significantly decreased in the berberine-induced group in vivo, and the metabolic activity of HepG2 cell in vitro could be increased by berberine. The metabolism parameters of lovastatin such as clearance (CL), maximum rate (V_max_) and Michaelis constant (K_m_) increased after treatment with berberine. Xin et al. [58] also studied the effects of berberine on the pharmacokinetics of midazolam and rhodamine 123 in rats. They showed that berberine could increase the C_max_ of midazolam while reducing its clearance rate and the volume of the distribution of midazolam. Similarly, berberine could increase the AUC of rhodamine 123 and raised the C_max_ of rhodamine 123 while at the same time shortening its volume of the distribution. Considering berberine being a substrate for P-gp, its interaction with the antifungal drug ketoconazole, which is an inhibitor of CYP3A and P-gp, is somehow expected. In oral co-administration (10 mg/kg ketoconazole with 60 mg/kg berberine) experiment in male rats, the average AUC and the C_max_ for ketoconazole were shown to increase to 215% and 449%, respectively [59]. Similar pattern was observed in female rats though to a less extent of drug interaction. In addition, the AUC and C_max_ for berberine was shown to increase to 173% and 142%, respectively, in drug combination study when compared to the berberine alone group. Interestingly, the intravenous coadministration (0.5 mg/kg ketoconazole and 0.8 mg/kg berberine) did not alter the pharmacokinetic properties of ketoconazole while the AUC of berberine increased to 254%. Berberine was further shown to inhibit (IC_50_ of 103 µM) the rat liver microsomes-mediated depletion of ketoconazole. Further examples with specific interest to the metabolic diseases are discussed in the following section.

## 4. The Physicochemical Modification Approaches

### 4.1. Potency Enhancement Using Novel Formulations

Entrapping berberine into solid lipid nanoparticles (SLNs) or berberine-loaded SLNs were studied for effect on lipid metabolism in the liver in *db*/*db* mice [60]. The maximum drug concentration in the liver was 20-fold higher than that in the blood. It also reduced fat accumulation and lipid droplet sizes in the liver; downregulated the expression of lipogenic genes, including fatty acid synthase (FAS), stearoyl-CoA desaturase (SCD1), and sterol regulatory element-binding protein 1c (SREBP1c); and upregulated lipolytic gene carnitine palmitoyltransferase-1 (CPT1). On the other hand, Wang et al. [61] used high pressure homogenization technique to prepare berberine nanosuspension that also incorporate D-α-tocopheryl polyethylene glycol 1000 succinate. In streptozotocin (STZ)-induced diabetic mice, treatment with this formulation (50 mg/kg) via an oral route for 8 weeks showed hypoglycaemic and total cholesterol (TC) and body weight reduction effects far better than the equivalent dose of either berberine or metformin (300 mg/kg).

With the hope of enhancing the oral bioavailability and anti-diabetic efficacy of berberine, Wang et al. [62] prepared an anhydrous reverse micelle delivery system through lyophilization of water-in-oil emulsions. In STZ-induced diabetic mice, administration of these berberine-loaded micelles could enhance the oral bioavailability of berberine by 2.4-fold, and the C_max_ by 2.1-fold with a 2-h time lag leading to a prolonged efficacy. Subsequently, the antidiabetic activity as a measure of blood glucose levels was better than unformulated berberine.

Zhaojie et al. [63] synthesised an amorphous solid dispersion of berberine that incorporate sodium caprate with enhanced absorption profile. This preparation named Huang-Gui Solid Dispersion (HGSD) showed a 3-fold increase in in vitro membrane permeation test using Caco-2 cell monolayer, a 4-fold increase in in situ rat intestinal perfusion and a 5-fold increase in vivo bioavailability in rats when compared to berberine or berberine tablets. Furthermore, oral administration (100 mg/kg) of the preparation has been shown to improve glucose and lipid metabolism in diabetic rats compared to pure berberine (100 mg/kg), berberine tablets (100 mg/kg) or metformin (300 mg/kg) treatment. With the aim of developing a more efficient berberine drug delivery system, Yu et al. [64] also synthesised a phytosomes loaded with berberine-phospholipid complex of nanoscale particle size by a rapid solvent evaporation method followed by a self-assembly technique. The resulting product with a negative surface charge, and excellent drug entrapment efficiency (∼85%) was shown to improve the oral bioavailability of berberine by 3-fold. Through oral route, the preparation (50 or 100 mg/kg for 4 weeks) could suppress the fasting glucose levels and improve the ability of systematic hyperlipidaemia metabolism of db/db diabetic mice. The activity of the synthesised compound both in uptake studies in vitro using human intestinal epithelial Caco-2 cells and antidiabetic efficacy (reduction in fasting blood glucose without change in insulin level) was better than unformulated berberine.

The antidiabetic effect of biofabricate berberine-coated nano-silver ameliorate was investigated for potential antidiabetic effects [65]. In acetaminophen-induced hepato-renal damages in diabetic rats, oral administration of this formulation (75 mg/kg, oral;ly (orally (p.o.)) could reduce the abnormally increased biochemical markers (aspartate aminotransferase (AST), alanine aminotransferase (ALT), lactate dehydrogenase (LDH), alkaline phosphatase (ALP), albumin, bilirubin and cholesterol, urea and creatinine) and lipid peroxidation (thiobarbituric acid reactive species (TBARS); while the disease-mediated diminished level of antioxidants (catalase (CAT), superoxide dismutase (SOD), ATPase and also Glucose 6-phosphatase (G6Pase) activities) along with morphological features of liver and kidney were improved. Other benefits include inhibition of the major proinflammatory regulator, the nuclear factor-factor-κB (NF-κB). The potential of selenium-coated nanostructured lipid carriers for enhancing the oral bioavailability and antidiabetic potential of berberine has been investigated by Yin et al. [66]. Administration of this preparation of the 160 nm particle size (50 mg/kg, p.o.) offered a sustained release of berberine with 6.63-fold improvement in oral bioavailability. Moreover, a better hypoglycaemic effect in STZ-induced diabetes model in rats than unformulated berberine was also recorded. Another good example of recent development in berberine’s delivery system was nanoemulsion-based delivery developed by Xu et al. [67]. With the small droplet size of about 30 nm and good stability, a lipolysis resistant formulation that protect berberine from CYP2D6 and CYP3A4-mediated intestinal metabolism was obtained. Cell transport and intestinal perfusion studies revealed a better bioavailability profile due to a 2-fold reduction in P-gl efflux and enhancement of permeability by 5.5-fold. In agreement with the cell transport studies, the oral bioavailability in rats increased by 212%. Consequently, the blood glucose level in diabetic mice could decrease by 3-fold when compared with unformulated berberine.

### 4.2. From Drug Combination to Structural Linkage with Other Bioactive Compounds

The rationale for combining one or more structural moieties with berberine came from studies that showed better antiobesity and/or antidiabetic outcome in drug combination studies. For example, Zhu et al. [68] studied the combination of berberine with resveratrol to show enhanced hypolipidaemic effects in high-fat diet (HFD)-induced obese mice. They have shown that combination of berberine (30 mg/kg/day, p.o.) with resveratrol (20 mg/kg/day, p.o.) could reduce the serum total cholesterol (TC) and LDL-C much more than either drugs alone. Similarly, combination studies with berberine (12 µmol/L or 20 µmol/L) and resveratrol (25 µmol/L) in 3T3-L1 adipocytes revealed a better inhibitory effect on lipid accumulation. Other important interaction observed in vitro was resveratrol increasing the intracellular level of berberine in hepatic L02 cells and their combination showed much pronounced LDL receptor expression in HepG2 cells than either of the compounds alone [66]. Since resveratrol is a known antioxidant compound with specific effects in cell signalling including adenosine monophosphate (AMP)-activated protein kinase (AMPK) and *nuclear* factor erythroid 2-related factor 2 (Nrf2) activation [69,70] and antidiabetic effect in various models [71,72,73,74,75,76], its combination studies with berberine appear to be a reasonable strategy.

Zhou et al. [77] studied the combination of berberine (72.6 mg/kg p.o.) and evodiamine (16.6 mg/kg p.o.) in cholesterol absorption in the intestine of HFD-induced hyperlipidaemic rats. They noted a reduction in the level of serum TC, triglycerides (TG), LDL-C, as well as hepatic TC in hyperlipidaemic rats after 4 weeks of treatment. Furthermore, the combination treatment downregulated the expressions of intestinal niemann-pick C1-like 1 (NPC1L1) and acyl coenzyme A: cholesterol acyltransferase 2 (ACAT2), and apolipoprotein B-48 (ApoB48) and the TC reduction by the two compounds were suggested to a synergistic action. The interaction between metformin and berberine in their bioavailability is somehow contradictory. Shi et al. [78] showed that berberine could decrease the C_max_, AUC from 0 to 4 h (AUC_0–4h_), and urinary and bile excretion, and increased the kidney tissue concentration of metformin in rats. On the other hand, coadministration of metformin increased the C_max_ and AUC_0–4h_ of berberine with no significant difference in pharmacokinetics parameters between coadministration and berberine-only groups. Metformin has also been shown to increase the kidney and liver concentrations and reduced the urinary and biliary excretion of berberine [78].

Zhang et al. [79] studied the interaction of baicalin with berberine for glucose uptake in 3T3-L1 adipocytes and HepG2 hepatocytes. While berberine was demonstrated to increase glucose consumption in 3T3-L1 adipocytes and HepG2 hepatocytes in a dose-dependent manner, baicalin (in doses up to 100 μmol/L) failed to do so. Interestingly, the combination of berberine and baicalin has a better profile of glucose uptake than those of either berberine or baicalin alone. This is further shown to have a trend of a synergetic effect on glucose uptake in a specific dose range. Antagonistic effect was also noted at a dose range in the presence of 10 μmol/L baicalin. Interestingly, the entire dose-response curves of berberine was shown to be shifted down in the presence of baicalin (100 μmol/L), and baicalin antagonised the effect of berberine on glucose uptake in 3T3-L1 adipocytes. Since the antioxidant flavonoid baicalein is known to improve a range of abnormalities associated with diabetes [80,81,82,83,84,85], its coadministration with berberine appear to have a favourable outcome in the specified dose range.

**Figure 3:**
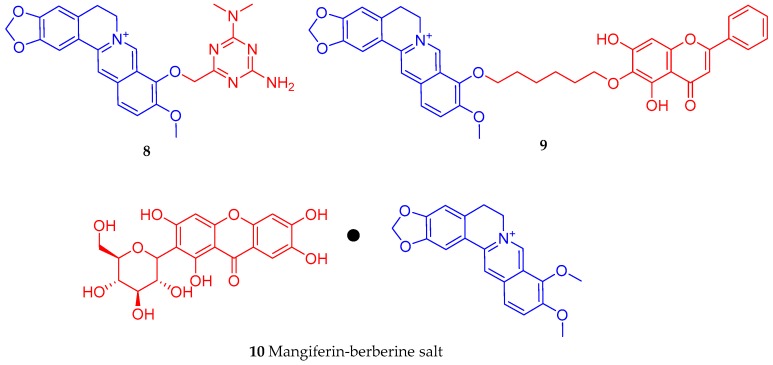
Some examples of berberine conjugates with bioactive compounds.

By using sodium methylate catalysis, Cao et al. [86] synthesized a triazine ring structural moiety product (**8**) from berberine and metformin. The compound (10–25 µmol/L) was shown to suppress inflammation as evidenced by the levels of cyclooxygenase-2/prostaglandin-E2 (COX-2/PEG-2) in lipopolysaccharide (LPS)-stimulated RAW264.7 cells. In INS-1 β-cells, the compound also showed an increase in insulin secretion by up to 156%. Another approach in berberine derivatisation was by making a conjugate (**9**) with baicalein (Figure 3). Following the conversation of berberine to berberrubine under microwave irradiation at 160 °C, Hao et al. [87] synthesised the 9-*O*-derivatives intermediate (9-(6-bromoethyl)berberine hydrochloride) by reacting with 1,6-dibromoethane which intern interact with baicalein under microwave irradiation reactions. In 3T3-L1 adipocytes, the hybrid berberine-baicalein compound (**9**) had an activity profile better than either berberine or baicalein alone. The compound up-regulated the expression of the adipose triglyceride lipase (ATGL) gene while down-regulating the mRNA expression of SREBP-1c, fatty acid synthase (FAS), tearoyl-CoA desaturase 1 (SDC1), and acetyl-CoA carboxylase (ACC) in 3T3-L1 adipocytes. Hence, an increase in lipolysis and decreased fat accumulation appears to be a mechanism of action for the compound.

The synthesis approach by Wang et al. [88] was based on the mangiferin-berberine salt formation (**10**) via ionic bonding of mangiferin and berberine at an equal molecular ratio (Figure 3). The rationale was based on the known antidiabetic effect of the xanthone glycoside mangiferin as antihyperlipidaemic [89,90,91,92,93,94,95], antidiabetic [96,97,98,99], and antioxidant [100,101,102] properties among others. In HepG2 cells, the salt increased the level of phosphorylated AMPKα (Thr172)/p-ACC (Ser79) and CPT1 activity and suppressed the oleic acid-induced lipid accumulation and upregulation of lipogenic genes. These AMPK-dependent activities were better than either berberine or mangiferin alone at an equal molar concentration and include enhancement of basal and insulin-stimulated glucose consumption and suppression of gluconeogenesis.

Jia et al. [103] prepared several metformin derivatives (Figure 3), including the above-mentioned novel berberine-metformin hybrid compound (**8**), which displayed anti-hyperglycaemic and anti-hyperlipidaemic effects in the experimental model of T2D rats. The compounds could reduce the perirenal and epididymal adipose tissue mass; modulate the lesions in perirenal adipose tissue; inhibit the protein expressions of peroxisome proliferator-activated receptor-γ (PPAR-Ɣ), CCAAT/enhancer binding protein α (C/EBP-α) and SREBP-1c as well as the mRNA expressions of lipogenic genes in white adipose tissue. Other effects reported for compound **8** include downregulation of the levels of pro-inflammatory cytokines in perirenal adipose tissue through suppression of the NF-κB; reduction in liver ectopic fat accumulation via regulatory mechanism on the protein expression levels of SREBP-1c and PPAR-α as well as the mRNA expression levels of lipogenic genes. Related to diabetes, the compound also inhibits hepatic gluconeogenesis by promoting the phosphorylation levels of AMPK-α and ACC, and down-regulating the mRNA expression levels of fructose-1,6-bisphosphatase (FBPase), G6Pase and phosphoenolpyruvate carboxykinase (PEPCK). In 3T3-L1 adipocytes, 8 also inhibits lipogenesis and lipid accumulation by modulating the protein expression levels of PPAR-Ɣ, C/EBP-α and SREBP-1 c as well as the mRNA expression levels of lipogenic genes [103]. Finally, the study by Fogacci et al. [104] explored the association between berberine and silymarin on serum lipids and fasting plasma glucose in human patients. Their meta-analysis of randomized, double-blind, placebo-controlled clinical trials with 497 subjects showed that the combination treatment exerted a positive effect on TC, high-density lipoprotein cholesterol (HDL-C), LDL-C, and fasting plasma glucose. Since the study showed better outcome for lipid and glucose profiles by coadministration of berberine and silymarin, further research on refining the combination through chemical synthesis approach should be encouraged.

### 4.3. Synthesis of Berberine Derivatives

**Figure 4:**
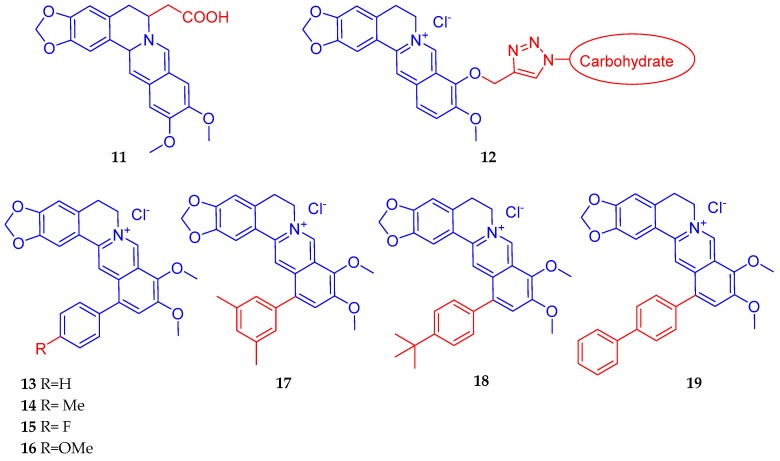
Berberine derivatives with potential antidiabetic effects [105,106,107].

By using in vitro glucose consumption activity in HepG2 cells, Wang et al. [105] synthesised a series of derivatives based on the structurally close relatives of berberine that showed hypoglycaemic activity via activation of the AMPK pathway. Their most active compound was compound **11** (Figure 4) which displayed an increased potency of 3.23-fold over berberine; 1.39-fold over metformin and 1.20-fold over rosiglitazone. Han et al. [106] synthesised some carbohydrate-modified berberine derivatives (**12**) (Figure 4) by assessing cytotoxicity and antidiabetic effect in HepG2 cells. In cytotoxicity assay, the mannose modified berberine derivative showed a potency (IC_50_ value) improvement of 1.5 times better than berberine. The mannose modified berberine derivative also showed potential anti-diabetic activity as shown by glucose consumption assay in vitro. Zhou et al. [107] synthesised eighteen novel 12-aryl berberine derivatives and evaluated them for their inhibitory effects on hypoxia-inducible factor (HIF)-1 transcription. As a result, seven 12-phenyl berberine analogues (**13–19**) (Figure 4) were identified with more potent inhibitory effect on HIF-1 transcriptional activity than berberine. Of these, compound (**19**) was the most potent with an IC_50_ value of 0.74 μM. The HIF-1 is a target for many diseases although cancer and inflammatory diseases have been recently highlighted as major diseases of interest. Accordingly, these compounds showed cytotoxicity against MCF-7 cancer cells, but whether the compounds offer a better profile in diabetes/hyperlipidaemia or obesity regulation remains to be established. In this direction, berberine is known to modulate HIF to improve insulin resistance in adipocytes [108], protect neuronal cells from ischaemia-induced damage [109,110], induce cytotoxicity in cancer cells [111,112,113,114], etc.

**Figure 5:**
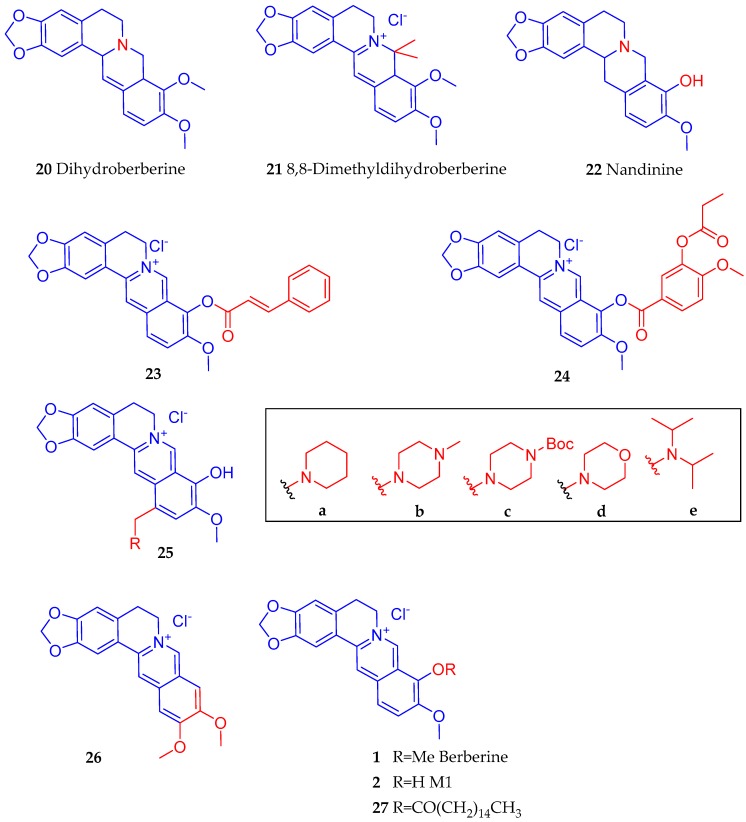
Further examples of berberine derivatives with antidiabetic promise.

To synthesise hypoglycaemic agents, Zhang et al. [115] employed a structure-activity studies on a serious of 9-*O* berberine ester derivatives: pyridine carboxylic acid berberrubine; nitrobenzoic acid berberrubine; cinnamic acid berberrubine; and benzoic acid berberrubine. In the glucose consumption assay in vitro, the compounds showed moderate activity while two compounds of the cinnamic acid berberrubine esters (**23** and **24**) (Figure 5) were identified as being more potent (increased activity of 33.6% and 27.4% respectively) than berberine at 5 µg/mL. Cheng et al. [116] synthesised a dihydroberberine (**20**) derivative 8,8-dimethyldihydroberberine hydrochloride (**21**) with improved bioavailability and oral efficacy in obese and diabetic mouse models. When tested in db/db diabetic mice at the dose of 15 or 50 mg/kg (p.o.), it was shown to be effective in suppressing adiposity, tissue and plasma triglyceride, fasting blood glucose and insulin resistance. The improved biological activity over dihydroberberine was also shown in vitro in L6 myotubes through inhibition of mitochondria respiration along with increased AMP:ATP ratio, activated AMPK and stimulated glucose uptake. In the study by Zhao et al. [117], which employed the macrophage-derived conditioned medium in 3T3-L1 adipocytes, dysregulation of adipokine production and activation of the I*κ*B kinase *β*/NF-κB pathway was induced. Using this model, they performed a comparative analysis of berberine and nandinine (tetrahydroberberrubine) (**22**) (Figure 5) on the regulation of insulin sensitivity in adipocytes. They showed that both berberine and nandinine could inhibit serine phosphorylation of insulin receptor substrate-1 induced by I*κ*B kinase *β* and increased tyrosine phosphorylation of insulin receptor substrate-1 to activate the phosphatidylinositol-3 kinase/protein kinase B (PI3K/Akt) pathway, which finally led to increased insulin-mediated glucose uptake. The compound (**22**) also increased the AMPK activity and could offer anti-inflammatory effect as it inhibits NF-κB activation. In mice, both berberine and nandinine at the doses of 100 or 200 mg/kg could abolish glucose intolerance and they enhance insulin sensitivity [117]. Further studies are, however, required to see which compound (nandinine vs. berberine) offer a better potential as a lead for the synthesis of a more potent derivative. By introducing various amino methyl groups into C-12 position of berberrubine, Li et al. [118] synthesised a series of 12-substituted aminomethyl derivatives. Although most of compounds showed moderate to good anti-diabetic activity, the enhancement of activity when compared to berberine was not that great. Compound **25b** (Figure 5) with an N-methyl piperazine-4-methyl group at C-12 was the most active compound in vitro in reversing insulin-resistance and stimulation of glucose transport in 3T3-L1 adipocytes and L6 myotubes. The insulin sensitization of this compound (**25b**) was 1.26-fold that of rosiglitazone [118].

Wang et al. [119] synthesised a series of new derivatives of berberine or pseudoberberine (**26**) (Figure 5) with potential activity on AMPK activation and upregulation of LDL receptor gene expression. The structural difference between 26 and berberine (**1**) is due to the dimethoxyl position: 9,10 in berberine and 10,11 positions in compound (**26**). Berberrubine (M1, 2), being an active metabolite of berberine, and hence berberine sometimes considered as a prodrug, Li et al. [120] synthesised derivatives with potential cholesterol-lowering activities with improved bioavailability. They identified compound **27** that incorporate the palmitate moiety at the 9-position with moderate LogP value and esterase hydrolysis rate for releasing M1 (**2**) in the blood. In hyperlipidaemic rats, its cholesterol-lowering effect when tested at 100 mg/kg was evident as it showed reduction in the blood CHO and LDL-C by 35.8% and 45.5%, respectively. It also exhibited a good safety in rats with no side effect on liver and kidney function.

## 5. General Summary and Conclusions

**Figure 6:**
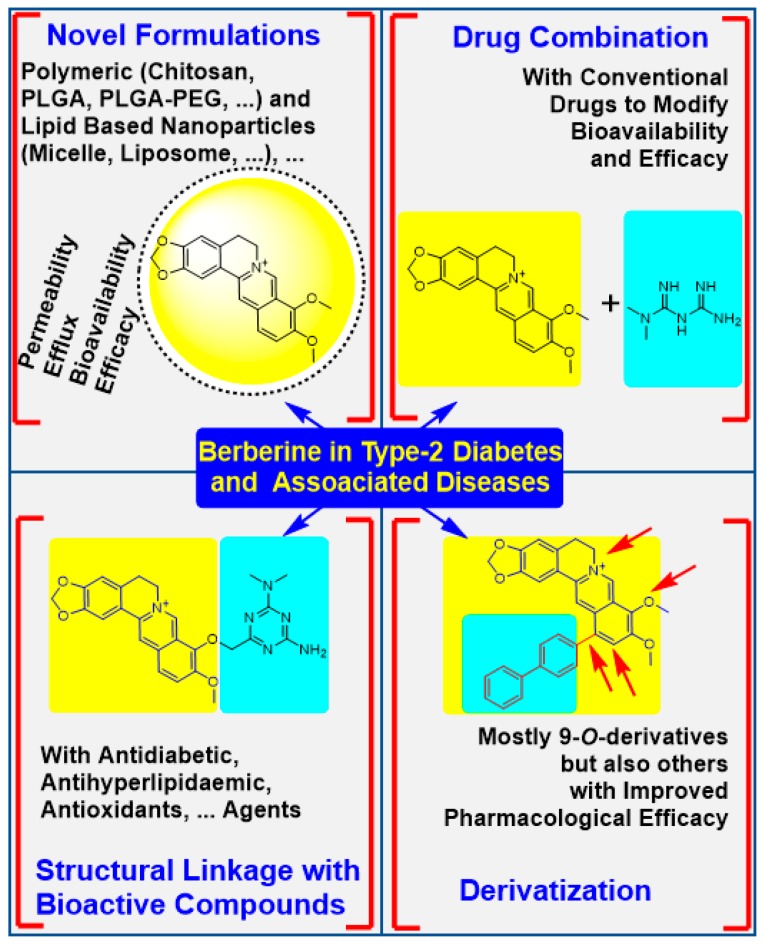
Physicochemical modification approaches for improvement of berberine’s pharmacology in T2D and associated disorders. Advances in formulation technology allowed berberine to be encapsulated alone or together with other agents while chemical modification include derivatization including hybrid formation with other pharmacologically active compounds. These approached have shown improvement in berberine’s absorption while reducing its efflux and degradation in intestinal tissues. The resulting improvement in bioavailability through formulation and efficacy improvement by chemical modification showed a better promise in experimental diabetes and other models of associated diseases.

As a natural compound, berberine is present in several medicinal plants that are still in use today. Its diverse pharmacological action through action on multiple target made berberine a classic example of natural products with potential to treat a plethora of disease conditions. Despite such potential, however, the bioavailability of berberine is far less than 1% due to poor absorption and efflux by P-gl. Accordingly, an explosive level of research has been devoted in the last two decades to improving the efficacy of berberine by various means. Considering the epidemic proportion of increase in the prevalence of diabetes and its major risk factor, obesity, in recent years, this article was designed to promote research in this area by scrutinising advances made in berberine pharmacology through physicochemical modifications. The four major areas of research presented in the paper are depicted in Figure 6. One major area of studies on berberine has been on improving bioavailability by using formulation technologies. An interesting review article for the general approach of berberine formulation is presented by Mirhadi et al. [121]. With respect to diabetes and associated diseases highlighted in the present paper, the formulations are designed in the first instance to increase the absorption of berberine wile suppressing the P-gl-induced efflux. Nanoparticles formulations that encapsulate berberine for sustained release and improved bioavailability include the use of polymeric natural (e.g., chitosan) and synthetic (PLGA, PLGA-PEG, etc.) agents. Others include a self-microemulsifying berberine-phospholipid complex of polyethylene glycol 1000 succinate (TPGS 1000) and SiO₂, phytosomes loaded with berberine-phospholipid complex, solid lipid nanoparticles, micelles, liposomes of various nature, etc. The nanoparticles of various charges from 30 nm to 160 nm sizes have been synthesised for evaluation of berberine’s efficacy improvement.

Another approach in berberine’s physicochemical modification was based on data from drug combination studies. While berberine is known to modulate the pharmacokinetics of various drugs, its own pharmacokinetic profile too is altered by other drugs. On the other hand, berberine has been shown to increase the efficacy of other antidiabetic agents such as metformin. Other combination studies include with the antioxidant compound resveratrol, an alkaloid evodiamine, and an antioxidant flavonoid, baicalin. It is this kind of study that has shown improved efficacy for berberine that give ground for structural modification by linking it with known bioactive compounds. This include a berberine hybrid with metformin (**8**), baicalein (**9**), and mangiferin (**10**). Various derivatives of berberine have also been synthesised and tested for their efficacy for diabetes and associated diseases. The 9-*O*-dervatives appear to dominate this initiative while others include 12-aryl berberine derivatives, the dihydroberberine and its metabolite (**2**) derivatives and others.

Studies using the human intestinal cell line monolayer in vitro (Caco-2 cells), ex vivo and animal studies have shown tremendous progress in the improvement of berberine bioavailability from the intestine via the above-mentioned formulation strategies. Inevitably, efficacy improvement by several-order of magnitude in lipid lowering and antidiabetic effects have been achieved. These formulations and synthetic berberine derivatives have been studied in vitro such as in adipocytes culture (3T3-L1 cells) to show increased pharmacological efficacy by suppressing lipogenic genes, increased lipolysis and/or improved lipid profile in vivo as shown by reduction in LDL-C, TC or increased level of HDL-C. On the other hand, in vitro glucose consumption assay in hepatic cells (HepG2 cells) and animal models such as STZ- and HFD-induced obesity/hyperlipidaemic models as well as db/db genetic model of obesity, diabetes, and dyslipidaemia were employed to show the antidiabetic potential of these compounds. As a mechanism of action, the known effect of berberine in cell signalling such as AMPK, PI3K/Akt, NF-κB, HIF, etc., or simply insulin, lipogenic and lipolytic, gluconeogenesis, inflammatory, and antioxidant pathways have been shown. These studies showing pharmacological efficacy should continue to identify the most potent derivatives with improved bioavailability and clinical efficacy.
